# An Audit on Patient Safety and Prescribing in Patients With a Learning Disability, Autism, or Both at a Scottish GP Practice

**DOI:** 10.1192/bjo.2022.481

**Published:** 2022-06-20

**Authors:** Amina Sardar, Dhruvashree Somasundara, Clare Carter, Christopher Weatherburn

**Affiliations:** 1 University of Dundee, Dundee, United Kingdom; 2 NHS Tayside, Scotland, United Kingdom

## Abstract

**Aims:**

The aim was to investigate the proportion of patients with a previous diagnosis of learning disability or autism who later successfully underwent an annual review of their prescribed anti-psychotic or anti-depressant. This audit was prompted after Public Health England announced that a significant number of adults with a learning disability, autism or both take a prescribed antipsychotic, an antidepressant or both without appropriate clinical indications (psychosis or affective/anxiety disorder).

**Methods:**

The sample included 23 patients from the practice who had received a diagnosis of learning disabilities, autism, or both by 12th October 2020. Of these, 12 patients had a record of at least 5 prescriptions of an anti-psychotic in the last 12 months and 20 patients had a record of at least 5 prescriptions of an anti-depressant within the last 12 months. The notes for these patients were reviewed in May 2021 in an effort to ascertain whether a medication review had been completed for these patients since May 2020. The review process included a phone call between the patient and the prescribing doctor to determine whether there any side effects were being experienced and to assess the need for the continuation of the prescription. The resulting data were recorded and analysed on Microsoft Excel.

**Results:**

Out of the 12 patients who had been prescribed an anti-psychotic, 10 had received a medication review within the last 12 months. From the 20 patients who had been prescribed an anti-depressant, 19 had undergone a review of their medication within the last 12 months.

**Conclusion:**

Review of anti-psychotics and anti-depressants prescribed to patients with a diagnosis of learning disability, autism, or both was overall positive with the majority of these patients receiving a medication review within 12 months. As a further recommendation, another audit can be done to explore whether these patients had an annual blood test done as increased cholesterol is a known side-effect of psychotropic drugs.

